# Collagen-Like Proteins (ClpA, ClpB, ClpC, and ClpD) Are Required for Biofilm Formation and Adhesion to Plant Roots by *Bacillus amyloliquefaciens* FZB42

**DOI:** 10.1371/journal.pone.0117414

**Published:** 2015-02-06

**Authors:** Xia Zhao, Yun Wang, Qianhan Shang, Yuyao Li, Haiting Hao, Yubao Zhang, Zhihong Guo, Guo Yang, Zhongkui Xie, Ruoyu Wang

**Affiliations:** 1 Gaolan Station of Agricultural and Ecological Experiment, Cold and Arid Regions Environmental and Engineering Research Institute, Chinese Academy of Sciences, Lanzhou, China; 2 Key Laboratory of Stress Physiology and Ecology in Cold and Arid Regions of Gansu Province, Lanzhou, China; 3 Key Laboratory of Desert and Desertification, Cold and Arid Regions Environmental and Engineering Research Institute, Chinese Academy of Sciences, Lanzhou, China; 4 Key Laboratory of Arid and Grassland Agroecology, School of Life Sciences, Lanzhou University, Lanzhou, China; Virginia Tech, UNITED STATES

## Abstract

The genes of collagen-like proteins (CLPs) have been identified in a broad range of bacteria, including some human pathogens. They are important for biofilm formation and bacterial adhesion to host cells in some human pathogenic bacteria, including several *Bacillus* spp. strains. Interestingly, some bacterial CLP-encoding genes (*clps*) have also been found in non-human pathogenic strains such as *B. cereus* and *B. amyloliquefaciens*, which are types of plant-growth promoting rhizobacteria (PGPR). In this study, we investigated a putative cluster of *clps* in *B. amyloliquefaciens* strain FZB42 and a collagen-related structural motif containing glycine-X-threonine repeats was found in the genes RBAM_007740, RBAM_007750, RBAM_007760, and RBAM_007770. Interestingly, biofilm formation was disrupted when these genes were inactivated separately. Scanning electron microscopy and hydrophobicity value detection were used to assess the bacterial cell shape morphology and cell surface architecture of *clps* mutant cells. The results showed that the CLPs appeared to have roles in bacterial autoaggregation, as well as adherence to the surface of abiotic materials and the roots of *Arabidopsis thaliana*. Thus, we suggest that the CLPs located in the outer layer of the bacterial cell (including the cell wall, outer membrane, flagella, or other associated structures) play important roles in biofilm formation and bacteria-plant interactions. This is the first study to analyze the function of a collagen-like motif-containing protein in a PGPR bacterium. Knocking out each *clp* gene produced distinctive morphological phenotypes, which demonstrated that each product may play specific roles in biofilm formation. Our *in silico* analysis suggested that these four tandemly ranked genes might not belong to an operon, but further studies are required at the molecular level to test this hypothesis. These results provide insights into the functions of *clps* during interactions between bacteria and plants.

## Introduction

Collagen is the most abundant protein presented in metazoans. It is the principal tensile element of vertebrate tissues such as tendon, bone, cartilage, and skin, where it occurs in the extracellular matrix [1], thus collagen is important for a broad range of functions, including tissue scaffolding, cell adhesion, cell migration, angiogenesis, cancer, tissue morphogenesis, and tissue repair [2]. The key structural feature of collagen is that it contains a unique repetitive amino acid sequence (Glycine-X-Y)_n_ pattern in helical proteins that form chains [3–5].

The occurrence of the (Glycine-X-Y)_n_ pattern has also been demonstrated in many bacterial proteins but their functions have been discussed only rarely [6]. Most proteins that contain collagen-related structural motif (CSM) patterns are distributed in the Firmicutes group (including mycobacteria and Gram-positive bacteria) and in some cases a single genome encodes more than one CSM-containing protein [7]. Interestingly, some recent reports suggest that collagen-like proteins (CLPs) may play important roles in the infectious processes of some Gram-positive human pathogens, such as *Bacillus anthracis*, [8–10], *Streptococcus* [11,12], and *Legionella pneumophila* [13]. Some studies have shown that CLPs are always involved in the colonization, motility, and location processes when bacteria interact with their hosts [14]. In *Streptococcus pyogenes*, streptococcal collagen-like protein-1 (*scl1*) is upregulated during the process of biofilm formation [15]. Moreover, the exact role of SCL1 in biofilm formation by Group A *Streptococcus* (GAS) is unknown, although Scl1-negative mutants exhibit a significantly decreased ability to form biofilms *in vitro* [16]. In *B*. *anthracis*, collagen-like protein 1 (*bcl1*) is a structural component of the filaments that cover the outer layer of the exosporium [8]. Recent studies have also shown that the CLP of *Legionella pneumophila* (*lcl*) could mediate its sedimentation and autoaggregation, and affect biofilm formation [17].

Although CSMs have been found in several pathogens, most CSM-containing bacteria are nonpathogenic. *B*. *amyloliquefaciens*, a Gram-positive plant growth-promoting *Rhizobacterium* (PGPR), was found to contain CSMs in the genomes of most of the sequenced strains, although *in silico* analysis suggested that there was a great variation in the CLP genes among different strains. The FZB42 strain, which was sequenced in 2007, contains four tandemly ranked CSM genes (RBAM007740, RBAM07750, RBAM007760, and RBAM07770) in its genome (GenBank accession no. NC009725 [18]). *B*. *amyloliquefaciens* can stimulate plant growth by secreting plant hormones, such as indole-3-acetic acid, and enhance mineral absorption by releasing phytase and siderophores into the environment [19]. Thus, antagonistic agents such as lipopeptide and polyketides are synthesized to combat phytopathogens [20–22]. The genome of FZB42 has a total length of 3928 kb and it contains 3693 open reading frames. The CLPs of *B*. *amyloliquefaciens* FZB42 share high homology with those of human pathogens in terms of their gene sequence, including *Streptococcus pyogenes* and *B*. *anthracis* [8,11,23].

Like most PGPR, FZB42 can form dense biofilms on the surfaces of root and it responds to root exudates by aggregating at root colonization sites to form stable biofilms in soils [24–26]. *Pseudomonas putida* can respond to root exudates in soils by converging at the root colonization site and establishing stable biofilms [25,26]. In addition, Gram-positive biocontrol agents such as *B*. *cereus* develop dense surface-associated populations, and a recent study linked biocontrol with the ability to form biofilms [27]. Several studies have focused on the biocontrol activity and colonization ability during interactions with plants, which are also likely to be related to biofilm formation [28–31].

In the model strain *B*. *subtilis* 168, biofilms are organized via an extracellular matrix, which predominantly comprises a protein component, TasA, and an exo-polysaccharide (EPS) component [32–34]. *B*. *amyloliquefaciens* FZB42 is taxologically similar to *B*. *subtilis*, but a major difference is that the latter lacks CLP genes [18]. The biological functions of CLPs in *B*. *amyloliquefaciens* have not been well studied, thus it not known whether they are functionally related to biofilm formation and root colonization. Thus, in the present study, we investigated the function of CLPs using site-directed mutagenesis and by analyzing the morphological variations in the colonies and cell shapes after each CLP gene was disrupted. The *clps* mutants exhibited a decreased ability to form biofilm and to adhere to the roots of *Arabidopsis thaliana*. Thus, we suggest that CLPs may contribute to the composition of the extracellular matrix, thereby affecting biofilm formation by *B*. *amyloliquefaciens* FZB42 during bacteria-plant interactions.

## Material and Methods

### Strains and growth conditions

The bacterial strains used in this study are listed in Table 1. *B*. *amyloliquefaciens* FZB42 was deposited as strain 10A6 in the culture collection of the Bacillus Genetic Stock Center (BGSC). For routine growth, bacteria were cultivated at 30°C in LB medium solidified with 1.5% agar. LB broth comprised 1% tryptone, 0.5% NaCl, and 0.5% yeast extract. MSgg broth comprised 0.5% glycerol, 0.5% glutamate, 5 mM Mops, 2 mM MgCl_2_, 700 μM CaCl_2_, 50 μg/mL tryptophan, 50 μg/mL phenylalanine, 50 μM FeCl_3_, 50 μM MnCl_2_, 2 μM thiamine, and 1 μM ZnCl_2_ in 100 mM morpholinepropanesulphonicacid buffer (pH 7.0) [33]. When necessary, antibiotics were used at the following concentrations: erythromycin (0.5 μg/mL), and ampicillin (100 μg/mL).

**Table 1:** Bacterial strains and plasmids used.

Strain or plasmid	Description	Source or reference
*Escherichia coli*		
DH5α		Lab strain
*Bacillus*		
FZB42	Wild-type isolate	BGSC 10A6
E-1	Δ*clpA*::Em^r^	This study
E-2	Δ*clpB*::Em^r^	This study
E-3	Δ*clpC*::Em^r^	This study
E-4	Δ*clpD*::Em^r^	This study
Plasmids		
pMD19-T	Cloning vector Amp^r^, lacZ＇	Takara Co. Ltd
pMUTIN4	Integration vector Em^r^, Amp^r^, lacZ＇	BGSC ECE139
pMUE-1	pMUTIN4 containing 0.59 kb insert of *clpA*	This study
pMUE-2	pMUTIN4 containing 0.656 kb insert of *clpB*	This study
pMUE-3	pMUTIN4 containing 0.832 kb insert of *clpC*	This study
pMUE-4	pMUTIN4 containing 0.872 kb insert of *clpD*	This study

### Construction of plasmids and strains

All of the plasmids used in this study are listed in Table 1. We used a basic integration vector, pMUTIN4, to construct knockout mutations of the *clp* genes by single crossover homologous recombination. Specific DNA fragments were amplified by PCR (ExTaq DNA Polymerase, Takara) using FZB42 chromosomal DNA as the template with the primers: *clpA*-F/*clpA*-R, *clpB*-F/*clpB*-R, *clpC*-F/*clpC*-R, and *clpD*-F/*clpD*-R (Table 2). The PCR products were cloned into the plasmid pMD19-T, which contains Hind III and BamHI restriction enzyme sites. These four restriction fragments were purified using a DNA gel extraction kit (OMEGA) and cloned into the integration plasmid pMUTIN4. These reconstructed plasmids were then transformed into FZB42 competent cells on coated plates and selected by monoclonal colony PCR with the antibiotic erythromycin.

**Table 2:** Primers used in this study.

Primer	Sequence (5′→3′)	Size of DNA sequence (bp)	Gene
ClpA-F	GAACTAAGAGATTGATGGGAC	590 bp	*clpA*
ClpA-R	CAGTCAGTACAGAGACTCTT	…	*clpA*
ClpB-F	AATCCGGAGACTTAACAGGC	656 bp	*clpB*
ClpB-R	GGAGCTACCGGACCAACTGGA	…	*clpB*
ClpC-F	CCAATGCCGCTGTTAA	832 bp	*clpC*
ClpC-R	GCTCACTGTTTACCCGCC	…	*clpC*
ClpD-F	CGACTCTTGGGATTACGAC	872 bp	*clpD*
ClpD-R	TCACGGCAGTGGGAAGAC	…	*clpD*

Competent cells of *B*. *amyloliquefaciens FZB42* were obtained by modifying the two-step protocol published by Kunst and Rapoport 1995 [35]. Cells were grown overnight in LB medium at 30°C (180 rpm) and diluted to an appropriate proportion (1:100) on the next day in 10 mL GCHE medium, which contained 0.1 M glucose, 0.005% w/v tryptophan, 0.04 M FeCL_3_/Na-citrate, 0.25% w/v potassium glutamate, 3 mM MgSO_4_, and 0.1% w/v casein hydrolysate. The cell culture was then incubated at 37°C with vigorous shaking (200 rpm) until the middle of the exponential growth period (OD_600_ = 1.4). The culture was diluted with an equal volume of GE medium (GCHE medium without casein hydrolysate) and the cells were then incubated for a further 1 h. Next, the culture was divided into five equal volumes and cells were harvested by centrifugation at 6000 rpm for 5 min. The cells were resuspended in 2 mL of transformation buffer, which contained 15 mM (NH4)_2_SO_4_, 80 mM K_2_HPO_4_, 45 mM H_2_KPO_4_, 35 mM sodium citrate, 1 mM EGTA, 25 mM glucose, and 30 mM Mg_2_Cl_2_, and 1 μg of DNA was added. After incubation at 37°C with shaking at 75 rpm for 20 min, 1 mL of LB medium containing a sublethal concentration (0.1 μg/mL) of the appropriate antibiotic was added. The cells were cultured with vigorous shaking for 90 min and plated onto selective agar plates.

### Biofilm formation assay

For the biofilm formation assays, cells were cultured from single colonies and then resuspended in 3 mL of LB at 37°C with shaking. When OD_600_ = 1.4, the cells were diluted to 1:100 in LB liquid medium containing an appropriate antibiotic in a 24-well plate and incubated at 30°C. To assess the colony morphology of the biofilms, 3 μL of the culture was plated onto MSgg medium at 30°C. Finally, we observed the biofilm morphology after 24 h and 72 h in liquid medium and on solid medium, respectively.

To quantify biofilm growth, we applied the crystal violet staining method in 96-well polystyrene plates (Thermo) [36–39]. Biofilm formation was assessed based on the cell adhesion morphology on the walls of the 96-well plate. An overnight culture (1:100) was added to 100 μL LB liquid medium in each well before incubating at 30°C. The following day, the cultures were stained with 40 μL 0.25% crystal violet for 15 min and washed three times with phosphate-buffered saline (PBS). Next, 200 μL of 95% ethanol was added to dissolve the biofilm for 15 min at room temperature before reading the OD_600_ values. Samples were analyzed in triplicate with at least three experiments.

### SEM of biofilms

*B*. *amyloliquefaciens* FZB42 cells were grown for 24 h on solid LB plates and smeared on a silicon slice on the object stage before SEM analysis. The samples were then imaged using a MIRA 3 scanning electron microscope at a magnification of 20000× with a beam voltage of 15 KV.

### Determination of hydrophobicity values

The cell surface hydrophobicity values were determined using a modified hexadecane method [16,40], where 5 mL of logarithmic-phase cells were centrifuged at 6000 rpm for 5 min, washed twice with PBS buffer, and resuspended in 5 mL of PBS, before recording the OD_600_values (A_0_). Next, 1 mL hexadecane was added to the suspension, which was vortexed for 1min, before allowing it to stand for 2 min to allow phase separation at room temperature, and the absorbance of the lower aqueous phase was read to determine the OD_600_ (A). The hydrophobicity values were calculated as follows: hydrophobicity value = [1—(A/A_0_)] × 100.

### Adherence to the surface of polystyrene and *A*. *thaliana* roots

A biofilm adherence assay was performed using polystyrene 96-well plates, where 100 μL of logarithmic-phase wild-type or *clp* mutant *B*. *amyloliquefaciens* FZB42 cultures were seeded without dilution into the wells and incubated for 24 h at 37°C in MSgg broth. After incubation, the wells were washed gently 2–3 times with 200 μL PBS. The wells were dried at 60°C for 1h and 200 μL 0.1% crystal violet was added to each well for 30 min to allow staining, followed by three washes with 200 μL PBS and solubilization in 200 μL ethanol:acetone (80:20, vol/vol). The OD_600_ values were determined using a Nanodrop 2000c reader (Thermo). The samples were analyzed in triplicate in at least six experiments.

A modified pour plate method was used to assay the amounts of bacterial colonies that adhered to the roots of *A*. *thaliana* [41]. The seeds of *A*. *thaliana* ecotype Columbia-0 were surface sterilized in 70% ethanol for only 30 s and in 30% NaClO for 8 min. The sterilized seeds were germinated on an agar (0.7%) plate of basal MS medium that contained 3% sterile sucrose and grown at 24°C with 16 h illumination for 7 days. The seedlings were then dipped in diluted (1000:1) overnight cultures of the bacterial cells for 10 min with gently rotation. After washing three times with sterilized water, 10 seedlings were placed in a flask containing 30 mL sterilized water and 20 glass beads, before incubating with shaking at 30°C for 20 min. After standing for 15 min, 100 μL of the homogenate was analyzed using the smear plate method and incubated at 30°C for 24 h. Each adhesion assay was performed at least three times. Three replicates were plated for each dilution level (1:10, 1:100, and 1:1000) to enumerate the colonies.

For scanning electron microscopy (SEM) observation, *A*. *thaliana* roots were soaked in wild type and *clp* mutants cultures for 20 min respectively, and then washed with sterilized water for two times. The samples were fixed with 2.5% glutaraldehyde in 0.1 M phosphate buffer for 24 h, and then rinsed with 0.1 M phosphate buffer and pH 7.4 at 4°C three times for 30 min each. Afterwards dehydration through a gradient series of acetone solutions and finally 100% isoamyl acetate, was followed by critical point drying. Specimen were then mounted on stubs for SEM and examined with a MIRA 3 scanning electron microscope with a beam voltage of 10 KV.

### Bacterial sedimentation assays

For the bacterial sedimentation assays, cells were cultured in 3 mL LB in optical tubes at 37°C with shaking at 200 rpm. The next day, 1:100 cultures were added to antibiotic-containing LB medium and incubated with shaking at 37°C. When OD_600_ = 0.7, the suspension was retained after reading the initial OD_600_ value, where the suspension was allowed to rest at 22°C and the OD_600_ value was recorded at intervals of 1 h to assess the level of bacterial aggregation [42]. This experiment was repeated at least six times. To further assess the level of aggregation, we used crystal violet staining to observe cell-cell interactions by 1000× oil microscopy. To verify the experimental accuracy, this experiment was also repeated six times [43].

## Results

### Domain architecture of CLPs in *B*. *amyloliquefaciens* FZB42 genomes

**Fig 1:**
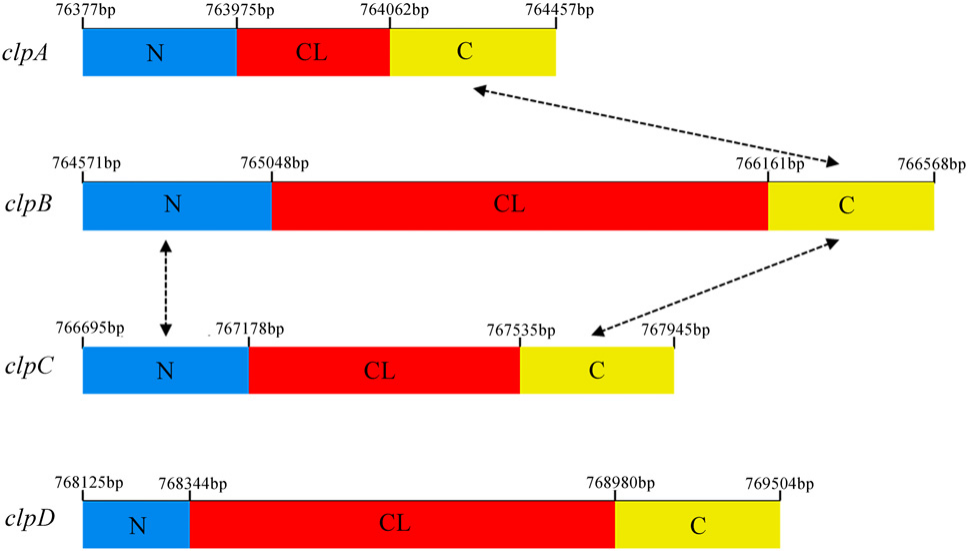
Homology between the nucleic acid sequences of putative domains in the four *clp* genes. Schematic representation to scale of the *clpA*, *clpB*, *clpC*, and *clpD* sequences from *B*. *amyloliquefaciens* FZB42. Translated Glycine-Xaa-Threonine repeats within the collagen-like domain (CL) are show by the red band; N, amino-terminal domains are shown by the blue band; C, carboxyl-terminal domains are shown by the yellow band. The proportion of shared homology between the terminal domains of each protein are shown by dotted arrowed lines. The positions of the four genes are registered according to the numbered nucleic acid bases in the genome of *B*. *amyloliquefaciens* FZB42. The sequence lengths of *clpA*, *clpB*, *clpC*, and *clpD* range from 763771 bp to 764457 bp, from 764571 bp to 766568 bp, from 766695 bp to 76945 bp, and from 768125 bp to 769504 bp, respectively.

To clarify the relationships between these proteins, we named the genes RBAM007740, RBAM07750, RBAM007760, and RBAM07770 as *clpA*, *clpB*, *clpC*, and *clpD*, respectively [18]. All four of these proteins comprise typical triplet (Glycine-X-Threonine) repeats (Fig. 1). The genes *clpA*, *clpB*, *clpC*, and *clpD* encode products of 228, 665, 416, and 459 amino acid residues, respectively. The collagen motif in *clpA* is much shorter than that in the others and it appears to be irregular with only 10 triplet repeats, and a third of the motif is replaced by other amino acids instead of threonine. There are longer Glycine-X-Threonine repeat units in the middle part of the peptide side-chains in *clpB*, *clpC*, and *clpD*, with two functionally unknown domains in the C-terminal and N-terminal.

Sequence alignments of the amino acid sequences of the four CLPs showed that the sequences shared 60% homology in the C-terminal domains of ClpA and ClpB, 52% homology in the N-terminal domains of ClpB and ClpC, and 30% homology in the C-terminal domains of ClpB and ClpC (Fig. 1). According to previous studies, we hypothesized that these four proteins may be surface proteins related to biofilm formation and we analyzed the roles of these domains in *B*. *amyloliquefaciens* FZB42.

### Inactivation of *clp* genes reduces biofilm formation

**Fig 2:**
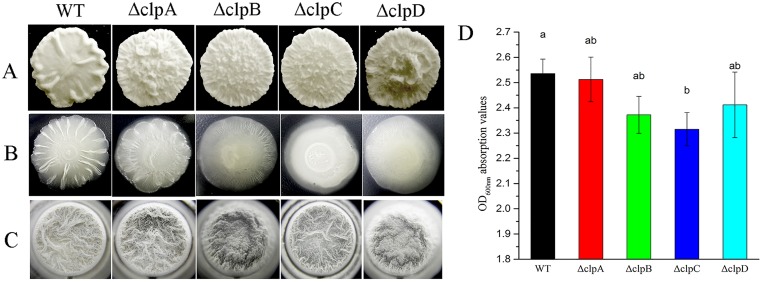
Variations in biofilm formation by the wild type and *clp* mutants. Biofilm formation by wild type and *clp* mutants in LB medium (A) and on MSgg medium plates (B). The images of colonies were obtained after incubation for 48 h at 37°C. (C) The biofilm images are top-down views of 96-well plates, which were obtained after incubation for 24 h at 37°C in MSgg liquid medium. (D) Quantitative spectrophotometric biofilm assay following crystal violet staining in MSgg medium. Analysis of variance detected a significant main group effect between the wild type and *clp* mutants (b, *P* < 0.05).

To confirm the roles of the four CLPs (ClpA, ClpB, ClpC, and ClpD) in biofilm formation, we constructed *clp* mutations by single crossover homologous recombination and different phenotypes were observed in terms of the biofilms produced by the wild-type and mutant strains (Fig. 2). The wild type and mutants produced different colony shapes on Luria-Bertani (LB) medium after cultivation for 48 h (Fig. 2A). The surfaces of the biofilms produced by Δ*clpA* and Δ*clpD* were more upheaval than those of Δ*clpB* and Δ*clpC*, whereas the center of each microcolony was relatively flat in the wild type. Thus, we used biofilm growth-specific minimal salts glycerol glutamate (MSgg) medium to determine whether CLP proteins are involved in the biofilm formation process (Fig. 2B). We found that the wild type had the highest capacity for biofilm formation, whereas the capacities of *clpA*, *clpB*, *clpC* and *clpD* were disrupted after 48 h cultivation on MSgg medium. The wild type and *clp* mutants exhibited different colony morphologies in liquid MSgg medium compared with liquid LB medium. The capacities of the *clpA*, *clpB*, *clpC*, and *clpD* mutants to form biofilms were reduced compared with the wild type after 24 h cultivation in liquid MSgg medium (Fig. 2C). Furthermore, the crystal violet staining method was also used in 96-well plates to quantify the biofilm formation ability of the different strains. The results showed that the biofilm formation capacity of *B*. *amyloliquefaciens* FZB42 wild type strain was the strongest whereas that of the *clpC* mutant strain was the weakest, where the *clpA*, *clpD*, and *clpB* mutants exhibited increasingly weaker biofilm formation capacities (Fig. 2D).

### *clp* mutants affect the cell surface matrix

**Fig 3:**
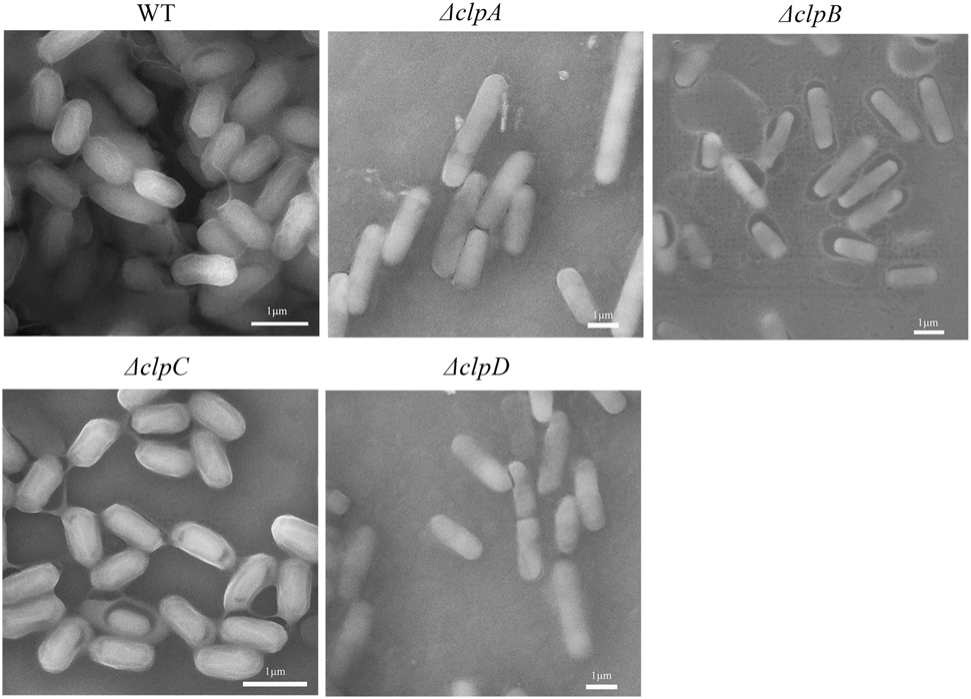
Scanning electron micrographs of wild-type and mutant biofilms. Cells were grown for 24 h on LB plates and smeared on a silicon slice on the object stage. The samples were then imaged using a MIRA 3 scanning electron microscope at a magnification of 20000× times with a voltage of 15 KV. Scale bar = 1 μm.

Scanning electron microscopy (SEM) was used to observe the bacterial cells of different *clp* mutants. We found that the cell surface of the wild type was enclosed by thick sheets, whereas pericellular sheets with abjunction or detachment were observed in the *clp* mutants (Fig. 3). We observed thread-like strands in the majority of the bacterial cells of the *clpA*, *clpB*, and *clpD* mutants, where the colonies formed comprised thin planar layers. Thus, we hypothesized that the CLPs may be components of the cell surface matrix, although further experiments are required to confirm this hypothesis.

We measured the cell surface hydrophobicity using the two-phase process method. The adsorption of bacteria is influenced by the charge, hydrophobicity, and structure of the cell surface (including the extracellular polysaccharide, flagellum, and pilus). One of the most important motive forces that facilitates bacterial nonspecific attachment to biological and nonbiological surfaces is determined by hydrophobicity [44–46]. The experimental data indicated that the hydrophobicity values of the *clp* mutants were reduced (Table 3). Table 3 shows that the wild type FZB42 strain had the highest hydrophobicity, i.e., 50.98 ± 3.5. By contrast, the hydrophobicity values of the Δ*clp* strains were much lower, where the *ΔclpC* strain was the lowest with 11.26 ± 4.3, followed by the *ΔclpD* strain with17.45 ± 2.6, *ΔclpA* with 24.89 ± 4.4, and *ΔclpB* with 24.29 ± 4.9. The hydrophobicity indices of *ΔclpA*, *ΔclpB*, *ΔclpC*, and *ΔclpD* on the cell surface decreased strikingly (100% for the wild type vs. 48.9% for *ΔclpA*, 47.6% for *ΔclpB*, 22.1% for *ΔclpC*, and 34.2% for *ΔclpD*), where those of *ΔclpC* and *ΔclpD* decreased the most obviously, i.e., by 77.9% and 65.8%, respectively. Therefore, *ΔclpA–D* modulated the hydrophobicity of the cell surface, thereby affecting the cell surface components.

**Table 3:** Cell surface hydrophobicity of wild type and *clp* mutants of *B. amyloliquefaciens* FZB42.

*B*. *amyloliquefaciens* FZB42 strain	Actual hydrophobicity value	Hydrophobicity index
wild type	50.98±3.5	100
Δ*clpA*	24.89±4.4	48.9
Δ*clpB*	24.29±4.9	47.6
Δ*clpC*	11.26±4.3	22.1
Δ*clpD*	17.45±2.6	34.2

The actual hydrophobicity values were calculated based on hexadecane binding, as described in the Methods section. The values (±SD) are representative of three experiments with four replicates. The hydrophobicity index represents the ratio of the actual hydrophobicity value for each strain relative to that of the isogenic wild type strain multiplied by 100.

### CLPs are involved in the autoaggregation of bacterial cells

**Fig 4:**
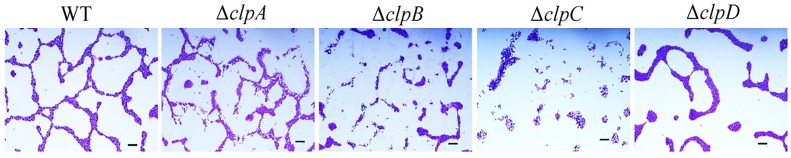
Micrographs of wild type and *clp* mutants obtained by optical microscopy. The autoaggregation phenotype was visualized by oil microscopy (Olympus CX31) at 100×/1.25 after incubation for 24 h. Each image is representative of four replicate experiments. Scale bar = 10 μm.

We obtained micrographs of the wild-type cells and *clp* mutant strains by optical microscopy after treating the cells with crystal violet staining, using a uniform field of vision with multiple repeats in each experiment. When the *ΔclpA*, *ΔclpB*, and *ΔclpC* genes were inactivated, the aggregations of the cells tended to be scattered to various degrees (Fig. 4). By contrast, the wild type and *ΔclpD* exhibited extensive cell-cell adhesion.

**Fig 5:**
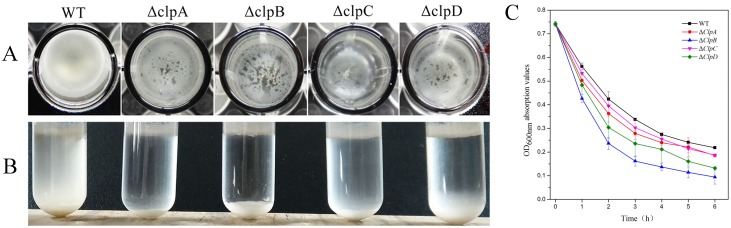
Roles of CLP proteins in bacterial aggregation. (A) Cells were grown in liquid LB medium for 72 h in 96-well plates. The images were obtained by viewing from the top to the bottom. (B) Cells viewed from front to back after standing for 10 h following 24 h incubation in glass test tubes. (C) Cell sedimentation assy. WT, Δ*clpA*, Δ*clpB*, Δ*clpC*, and Δ*clpD* bacteria were grown until OD_600_ = 0.7 and the bacterial precipitates were suspended by mixing, before the OD_600_ values were measured at 1 h intervals.

Interestingly, a floating biofilm was obtained in liquid LB medium after sedimentation following 72 h culture in 96-well plates (Fig. 5A). We also observed this phenomenon after 10 h standing following 24 h incubation in glass test tubes (Fig. 5B). Compared with the wild type, the floating biofilms produced by the *clp* mutants sank much faster. Therefore, we suggest that the CLPs are involved in cell-cell interactions or cell autoaggregation, and cell sedimentation experiments were used to test this hypothesis. As shown in Fig. 5C, the wild type and *clp* mutants were grown overnight, and the OD_600_ values were measured at 1 h intervals after the OD_600_ reached about 0.7 on the following day. The OD_600_ value of the wild type declined from 0.7 to 0.3 after 4 h, whereas those of Δ*clpA* and Δ*clpC* each decreased from 0.7 to 0.2 after 6 h. Remarkably, the OD_600_ values of the Δ*clpB* and Δ*clpD* cell sediments declined rapidly from 0.7 to 0.15 and from 0.7 to 0.1, respectively. In agreement with the results described above, the sinking cells of the *clp* mutants exhibited biofilms with a broken surface. These results suggest that the CLPs contribute to the promotion of inter-cell contacts and autoaggregation.

### CLPs can adhere to abiotic and root surfaces

**Fig 6:**
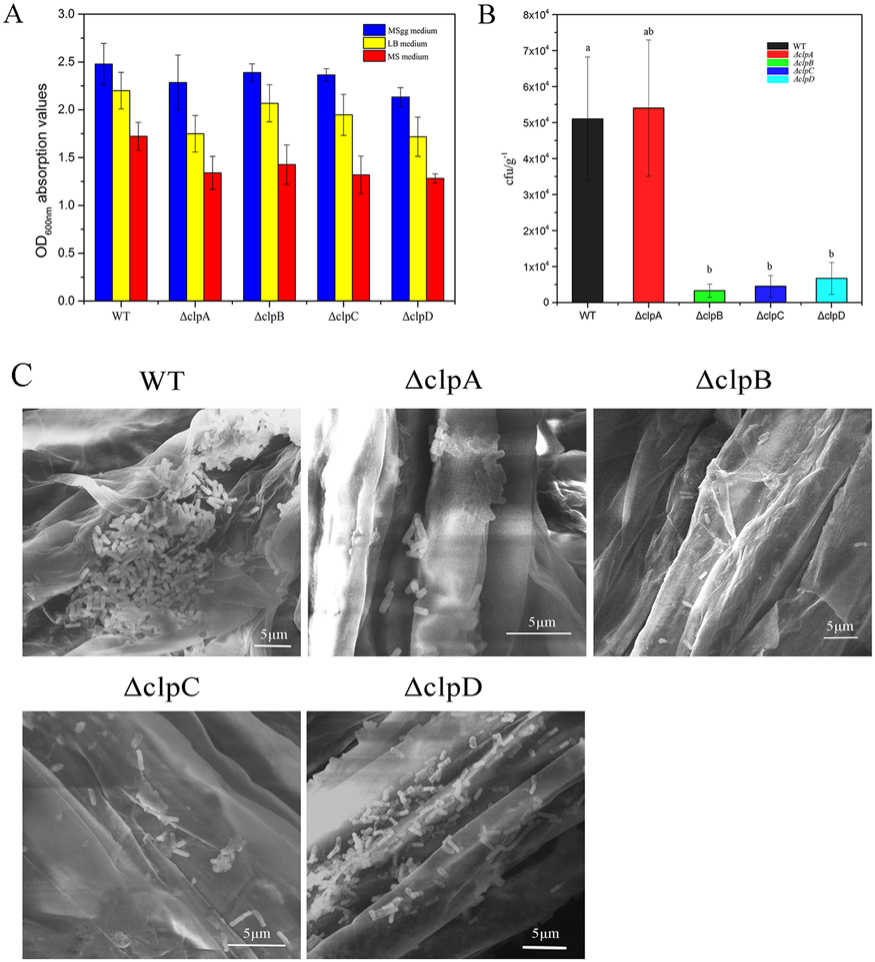
Adherence capacities of wild type and *clp* mutants on abiotic and root surfaces. (A) Adherence capacities of wild type and *clp* mutants on polystyrene surfaces. The OD_600_ values indicate the biofilm adherence capacities of the wild type, *clpA* mutant, *clpB* mutant, *clpC* mutant, and *clpD* mutant to polystyrene surfaces. The cells were grown in MSgg medium, LB medium, and MS medium, respectively. (B) Adherence capacities of wild type and *clp* mutants to the roots of *A*. *thaliana*. The experiments were performed five times and similar results were obtained. The values represent the means ± standard deviations based on 12 measurements. Analysis of variance detected a significant main group effect between the wild type and *clp* mutants (b, *P* < 0.05). (C) Scanning electron microscopy (SEM) of wild type and *clp* mutants adhere to the roots surface of *A*. *thaliana*. SEM images were taken 20 min after bacterial soaking. The samples were imaged using a MIRA 3 scanning electron microscope at 10 KV. Scale bar = 5 μm.

To further characterize the functions of CLPs, we assessed their adherence to the surfaces of abiotic materials. The capacity for biofilm adherence to polystyrene surfaces was analyzed using a crystal violet staining assay with the wild type and *clpA*, *clpB*, *clpC*, and *clpD* mutant strains in 96-well plates with MSgg, LB, and Murashige-Skoog (MS) media (Fig. 6A). When the *clp* genes were inactivated, the biofilm adherence capacity was reduced to different degrees compared with the wild type in each medium. The *clpC* mutant exhibited the lowest absorption values, followed by the *clpB* mutant strain, whereas those of the *clpA* and *clpD* mutant strains declined only slightly. *B*. *amyloliquefaciens* FZB42 is a PGPR strain, thus we determined whether CLPs can also adhere to the roots of *A*. *thaliana*. To measure the adherence capacity, we used 7-day-old *A*. *thaliana* seedlings, which were dipped in diluted bacterial cultures of the wild type and *clp* mutants, before determining the number of colony-forming units on the roots. We found that the adherence capacities of the *clpA*, *clpB*, and *clpD* mutants were significantly lower than those of the wild type and the *clpA* mutant (Fig. 6B). The difference between the *clpA* mutant and the wild type was not significant. The SEM micrographs of *A*. *thaliana* root surface also showed that wild type and *clpA* mutant cells were largely adhere to root (Fig. 6C). While, there were only few cells of *clpB* and *clpC* mutants were adhere to root.

## Discussion

### Potential functional domains in CLPs

Bacterial CLPs all share a typical collagen domain with a repeating amino acid sequence [6]. The triplet repeat (Glycine-X-Threonine)_n_ was identified from CLPs in the genome of *B*. *amyloliquefaciens* FZB42. It has been reported that Scl1 and Scl2 are organized into “lollipop-like” structures in streptococci, which are similar to the collagenous domains in human proteins [47]. The CLPs in *E*. *coli* O157:H7 also form a “dumb-bell” shape with two globular domains joined by a hinged stalk [48]. Similarly, Ayumi and Yu purified recombinant CLPs (Scl1 and Scl2) from *Streptococcus pyogenes*, which comprised an N-terminal globular domain V followed by the collagen triple-helix domain CL, with an array of dimeric head (V)-to-head V-CL-CL molecules [49]. However, a globular C-terminal domain was located at the distal end of the filaments, thereby forming a robust permeability barrier or shield around the bacterial spore [50].

**Fig 7:**
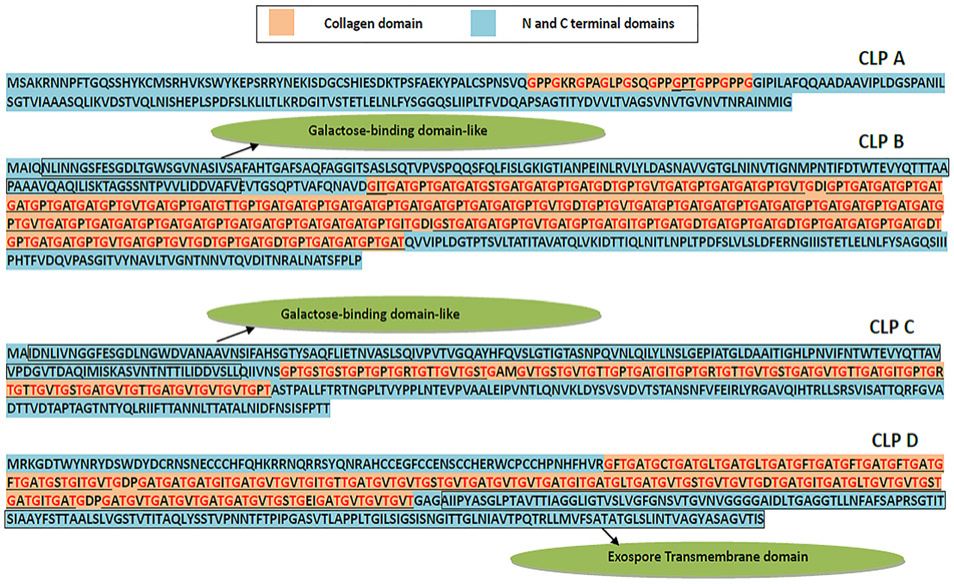
Architecture and amino acid sequences of ClpA-ClpD in *B*. amyloliquefaciens FZB42. The first position of each Glycine-Xaa-Yaa repeat is shown in red. The repetitive sequences of Gly-Xaa-Thr are underlined. The results of the *in silico* analysis of the potential domains are shown in blue with rectangular boxes and the descriptions are shown in green ellipses.

Online INTERPRO analysis (http://www.ebi.ac.uk/interpro/) showed that a predicted galactose-binding domain is present in the N-terminals of ClpB and ClpC (Fig. 7). There is also a potential domain in the C-terminal of *clpD*, which is similar (99% identity) to the C-terminal of BclB, a protein found in the filaments that cover the outer layer exosporium of *B*. *anthracis*. Similar to the BclB protein [51], *clpD* was shown by TMHMM (http://www.cbs.dtu.dk/services/TMHMM/) to contain three transmembrane domains in its C-terminal. The N-terminal and C-terminal sequences of the *clp* domain were also identified. Given the high homology of *clpA*, *clpB*, *clpC*, and *clpD* according to the present study, these CLPs may share a similar structural assembly mechanism or perform a single function synergistically, but this requires further clarification.

### *B*. *amyloliquefaciens* FZB42 and biofilm formation

We designed an experiment where all four *clp* genes, i.e., *clpA*, *clpB*, *clpC* and *clpD*, were inactivated separately by site-directed mutagenesis. After observing the biofilms formed by these four mutant strains, we showed that biofilm formation was generally attenuated, especially in the *clpB* and *clpC* mutants, which formed thicker colonies with more irregular surfaces (Fig. 2). In nature, bacteria often exist as sessile communities called biofilms, which are important structural features where the cells are bound together by an extracellular matrix [52]. EPS and the protein TasA are known to contribute to the major extracellular matrix in different ways in *B*. *subtilis* [32]. However, genome analyses have shown that not all of the sequenced strains of Bacillus possess CLP genes, whereas strains of other *Bacillus* species such as *B*. *cereus*, *B*. *licheniformis*, *B*. *anthracis*, and *B*. *amyloliquefaciens* possess CLP genes in their genomes. Interesting, although *B*. *anthracis* is the human pathogen among these species, many CLPs have been found in pathogenic bacteria and they are defined as proteins that facilitate attachment to host tissues by bacterial pathogens, thus they are involved in the infection and colonization process [53]. Previously, we found that novel biofilm formation-related proteins, CLPs, were present as multiple copies in many strains of *B*. *amyloliquefaciens*. Thus, we investigated whether the CLPs found in PGPR play roles in the interactions between bacteria and root surfaces. First, we determined whether CLPs are located in the surface regions of bacterial cells in *B*. *amyloliquefaciens* strain FZB42 before assessing whether CLPs play roles in biofilm formation. We found that the roles of CLPs in biofilm formation are consistent with previous studies of CLPs in *Streptococcus pyogenes*, *B*. *anthracis*, and *Legionella pneumophila* [8,10,13,16].

### Roles of CLPs in the extracellular matrix

In previous studies, CLPs (BclA and BclB) were reported to be surface proteins in *B*. *anthracis* that contribute mainly to exosporium surface proteins [10]. BclA is expressed on the surface of spores and in sporulating cells, and it is an important structural component of the filaments that cover the outer layer of the exosporium [8]. Scl1 and Scl2 are CLPs in *Streptococcus pyogenes* that mediate GAS-cell surface hydrophobicity and that contribute to biofilm formation [16,54]. In the present study, we found that the extracellular matrix was separated slightly from the cells when *clpB* and *clpC* were inactivated (Fig. 3). The colonies produced planar layers in the *clpA* and *clpD* mutant strains according to our SEM observations (Fig. 3). The shapes of the cells of the *clpA*, *clpB*, *clpC*, and *clpD* mutants were morphologically similar to that of the *eps* gene mutants in *B*. *subtilis* 168, which were also observed using SEM by Branda [32]. By contrast, the wild-type cells were covered by a thick envelope in *B*. *amyloliquefaciens* FZB42. Moreover, we found that ClpD shared high homology with BclB based on its amino acid sequence. This suggests that CLPs may be important for maintaining the structure of the bacterial extracellular matrix.

Interestingly, the wild-type cells of *B*. *amyloliquefaciens* FZB42 exhibited a three-dimensional shape with a “jelly-like” coating over the extracellular matrix, whereas the *clpB* mutants exhibited a “flattened” and “long rod-like” shape (S1 Fig.). The shapes of the *clpA*, *clpC*, and *clpD* mutant cells are not shown in this study. Given this evidence, we conclude that CLPs contribute to biofilm formation, but we also hypothesize that they construct the supporting framework for bacteria by forming a “cross-linked” envelope. This hypothesis will be tested in our future research.

### Autoaggregation of CLPs in the “cell-cell” interactions

In this study, when CLPs were inactivated in FZB42, the bacterial colonies tended to be more “scattered” (Fig. 5A). In addition, the important role in cell auto-aggregation played by CLPs was supported by the results of the sedimentation experiments (Fig. 5B). Based on these results, we hypothesized that CLPs might be cell surface components. In agreement, we found that the hydrophobicity values of the cell surfaces were lower in the *clp* mutant strains (Table 3). In addition, Fig. 6 shows that CLPs have important effects on the capacity to adhere to abiotic surfaces. These results are consistent with previous studies, which showed that activation of CLPs can promote several biological functions, including cell adhesion and autoaggregation [55,56]. These results led us to consider the relationship between CLPs and biological surfaces. *B*. *amyloliquefaciens* FZB42 is a PGPR strain, thus the roots of *A*. *thaliana* were used in adhesion experiments. Interestingly, the adherence capacity was reduced significantly when *clpB*, *clpC*, and *clpD* were inactivated individually compared with the *clpA* mutant and the wild type. Thus, these results showed that CLPs of *B*. *amyloliquefaciens* FZB42 are likely required for the plant-bacterial adhesion on the roots surface of *A*. *thaliana*.

Furthermore, it has been reported that the extracellular matrix (such as the pilus, flagella, and membrane proteins) of some bacteria can affect “cell-cell” interactions by mediating cell autoaggregation and adhesion [57,58]. Thus, we tentatively suggest that CLPs may be components of the outer membrane protein or flagella proteins. However, our experimental results demonstrate that CLP proteins are probably components of the extracellular matrix of *B*. *amyloliquefaciens* FZB42 and they have major effects on the physical properties of the bacterial cell surface.

## Supporting Information

S1 Fig Cell surface morphology of the wild type and *clpB* mutant. The cell micrograph on the left shows the wild type and that on the right shows the *clpB* mutant after biofilm growth for 24 h, where the images were captured using a MIRA 3 scanning electron microscope. The red arrows indicate the ‘jelly-like’ matrix. Scale bar = 1 μm.
(TIF)
