# Supplementary figures and images for: Collagen-Like Proteins (ClpA, ClpB, ClpC, and ClpD) Are Required for Biofilm Formation and Adhesion to Plant Roots by Bacillus amyloliquefaciens FZB42
Source: PLoS One. 2015 Feb 6;10(2):e0117414. doi: 10.1371/journal.pone.0117414 (PMC4319854; doi:10.1371/journal.pone.0117414)

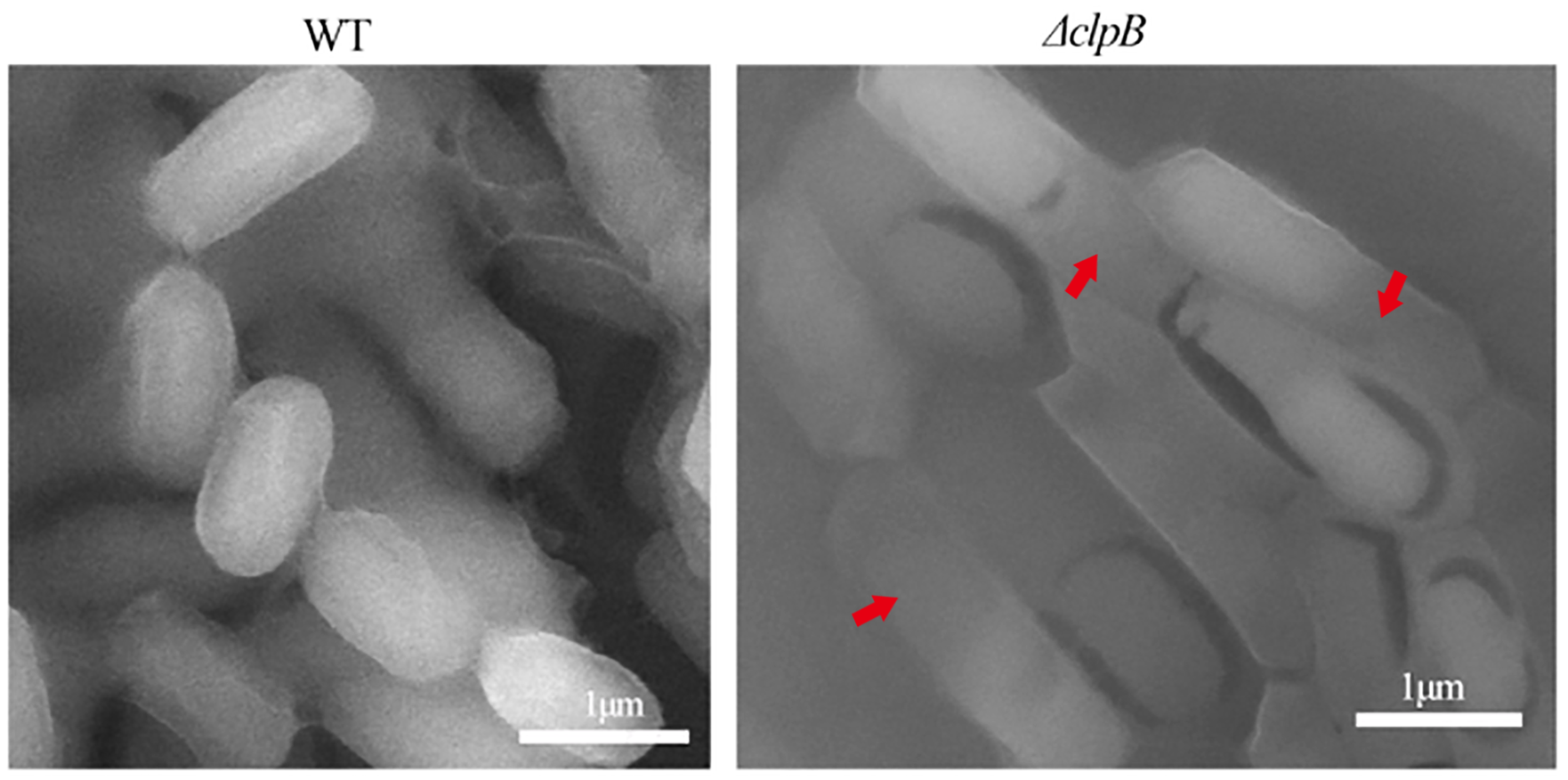

Supplement: S1 Fig — The cell micrograph on the left shows the wild type and that on the right shows the clpB mutant after biofilm growth for 24 h, where the images were captured using a MIRA 3 scanning electron microscope. The red arrows indicate the ‘jelly-like’ matrix. Scale bar = 1 μm. (TIF) [file pone.0117414.s001.tif]
